# Do personality traits moderate the manifestation of type 2 diabetes genetic risk?^☆^

**DOI:** 10.1016/j.jpsychores.2015.07.003

**Published:** 2015-10

**Authors:** Iva Čukić, René Mõttus, Michelle Luciano, John M Starr, Alexander Weiss, Ian J Deary

**Affiliations:** a Centre for Cognitive Ageing and Cognitive Epidemiology, University of Edinburgh, UK; b Department of Psychology, University of Edinburgh, UK; c Department of Psychology, University of Tartu, Estonia; d Geriatric Medicine Unit, Western General Hospital, Edinburgh, UK

**Keywords:** Personality, Polygenic risk, Type 2 diabetes, Gene–trait interaction, HbA1c, Cognitive ability, Conscientiousness, Agreeableness, Openness

## Abstract

*Objective*. To test whether personality traits moderate type 2 diabetes (T2D) genetic risk. *Methods*. Using a large community-dwelling sample (*n* = 837, *M*_age_ = 69.59 ± 0.85 years, 49% males) we fitted a series of linear regression models predicting glycated haemoglobin (HbA1c) from T2D polygenic risk — aggregation of small individual effects of a large number of single nucleotide polymorphisms (SNPs) — and five personality traits. We tested the main effects of personality traits and their interactions with T2D polygenic risk score, controlling for age and sex. The models in the final set were adjusted for cognitive ability, highest educational qualification, and occupational class. *Results*. Lower levels of openness were associated with heightened levels of HbA1c (*β* = − 0.014, *p* = .032). There was a significant interaction between T2D polygenic risk score and agreeableness: lower agreeableness was related to a stronger association between T2D polygenic risk and HbA1c (*β* = − 0.08, *p* = .021). In the model adjusted for cognitive ability, the main effect of openness was not significant (*β* = − 0.08, *p* = .057). The interaction between agreeableness and T2D polygenic risk was still present after controlling for cognitive ability and socioeconomic status indicators, and the interaction between conscientiousness and polygenic risk score was also significant: lower conscientiousness was associated with a stronger association between T2D polygenic risk and HbA1c levels (*β* = 0.09, *p* = .04). *Conclusions*. Personality may be associated with markers of diabetes, and may moderate the expression of its genetic risk.

## Highlights

• We tested whether personality moderates type 2 diabetes (T2D) genetic risk.
• Lower O was associated with higher HbA1c before adjusting for cognitive ability.
• Association between T2D polygenic risk and HbA1c was stronger in low A and low C.
• Adjusting for socioeconomic status did not alter the relationships.
• Personality may influence the genetic tendency to T2D.

## Introduction

Diabetes mellitus is one of the biggest contemporary disease burdens [1]. It is a major risk factor for health complications, including heart disease and stroke, blindness, kidney and nervous system disease, limb amputations, and increased risk of death [2]. In 2011 there were 366 million people with diabetes worldwide, approximately 90% of whom had type 2 diabetes and this number is expected to rise to 552 million by 2030 [3,4]. Given the personal and social costs associated with diabetes, it is imperative to find ways to identify at-risk individuals early, and to engage them in prevention strategies [4,5].

Behavioural factors such as unhealthy diet and physical inactivity, as well as obesity, older age, insulin resistance, and the metabolic syndrome are all commonly recognized risk factors for type 2 diabetes [6]. In addition to these, genetic risk also contributes to type 2 diabetes development [7]. About 10% of the genetic risk is explained by known common genetic variants [8] with the remainder being attributable to small individual effects of a large number of single nucleotide polymorphisms (SNPs) [9]. These small effects may not be individually detectable by current GWAS studies [9], but they may be aggregated across thousands of SNPs to quantify diabetes genetic risk [10–12]. The manifestation of the genetic risk may be moderated by non-genetic factors. For example, there is evidence that dietary habits may interact with the genetic factors predisposing to type 2 diabetes [13–16]. Psychological factors, including those related to the aforementioned dietary habits and depression, could moderate the expression of diabetes among those who are at high genetic risk.

Mõttus et al. recently found that childhood cognitive ability moderates the relationship between polygenic risk for type 2 diabetes and late life HbA1c levels: participants at a given level of genetic risk with lower cognitive ability at age 11 years were more likely to have heightened HbA1c levels at age 70 than participants with higher childhood cognitive ability [17]. This behavioural trait by genetic risk interaction offers an insight into potential mechanisms by which psychological characteristics may influence health outcomes.

Besides cognitive abilities, other stable behavioural characteristics such as personality traits described by the Five Factor Model [18,19] have been associated with diabetes in both cross-sectional [20,21] and longitudinal [22] studies. More specifically, in one cross-sectional study, participants with diabetes had lower conscientiousness, agreeableness and openness [20]. In another study, participants with diabetes had higher levels of neuroticism [21]. However, in a longitudinal study, higher levels of neuroticism were associated with lower risk of developing type 2 diabetes [22]. In a pooled analysis of five cohorts, lower conscientiousness was related to higher diabetes incidence and mortality [23]. Furthermore, personality has been shown to influence a wide range of behavioural and physiological diabetes risk factors. For example, higher neuroticism and lower openness are related to obesity, high triglycerides, hypertension, and elevated blood glucose, all of which are components of the metabolic syndrome [24,25]. Along with higher neuroticism and lower openness, higher levels of extraversion have been related to aspects of diabetes-prone lifestyle such as unhealthy dietary habits and low levels of physical activity [26,27]. In addition, lower agreeableness has been associated with higher alcohol intake [28]. It is, then, possible that personality trait may influence diabetes by moderating its genetic risk in a way similar to cognitive abilities as shown by Mõttus et al. [17].

Specifically, we hypothesized that personality traits moderate whether genetically at-risk individuals have higher levels of glycated haemoglobin, a diagnostic tool for diabetes mellitus. This hypothesis is based on the direct and indirect — i.e., via other risk factors, associations between personality traits with diabetes, as well as on the recent study [17] showing that higher cognitive ability is protective among participants who are at high genetic risk for diabetes.

## Method

### Sample

Participants were community-dwelling members of the Lothian Birth Cohort 1936 (LBC1936) — a follow-up study of the Scottish Mental Survey 1947 (SMS1947). On June 4, 1947, nearly all children born in 1936 and attending school in Scotland (*n* = 70,805; 35,809 boys) sat the Moray House test of intelligence. Between the years 2004 and 2007, participants from Edinburgh and the Lothians region were identified through the Community Health Index and media advertisements. Of 3810 identified participants, 3686 were identified to take part in the follow-up study. In total, 2318 responses were received, of which 1226 met the eligibility criteria to take part in the study. The final number of tested LBC1936 participants was 1091 [29]. Participants who dropped out of the study during the follow-up time were, on average, of lower intelligence and poorer health status than those assessed in the follow-up, but these difference were relatively small [29]. Full details on the recruitment and testing procedures are provided elsewhere [30]. Of the initial 1091 LBC1936 members, complete data on age, sex, personality traits, HbA1c, and genetic risk were available for 837 participants (*M*_age_ = 69.59 ± 0.85 years, 48.7% males). All participants provided written informed consent. Ethical approval was obtained from the Ethics the Multi-Centre Research Ethics Committee for Scotland.

### Measures

#### Personality

Personality was assessed using the 60-item NEO Five-Factor Inventory (NEO-FFI), a valid and reliable instrument designed to assess the five personality domains — neuroticism, extraversion, openness, agreeableness, and conscientiousness — of the Five Factor Model [31]. Approximately 50% of the variance in personality traits can be accounted for by genetic influences [32].

#### Glycated haemoglobin

Glycated haemoglobin (HbA1c) is typically used as an indicator of long-term blood glucose levels [33] and as a diagnostic criterion for diabetes mellitus [34]. About 75% of the variation in the levels of Hba1c is due to genetic influences [35]. The HbA1c levels were analysed from blood taken during participants' visit to the clinic, and were treated as a continuous variable.

#### T2D polygenic risk

All participants underwent genome-wide genotyping, conducted by the Genetics Core Laboratory at the Wellcome Trust Clinical Research Facility, Western General Hospital, Edinburgh, UK, using the Illumina Human 610-Quadv1 Chip (Illumina Inc., San Diego, CA, USA). The quality control procedures included checks for gender discrepancies, individual relatedness and non-Caucasian descent, and are fully described elsewhere [36].

The polygenic risk score for type 2 diabetes was calculated for each member of the LBC1936 following a previously published meta-analysis on the association between type 2 diabetes and approximately 121,000 SNPs. The meta-analysis comprised 34,840 participants with type 2 diabetes diagnosis and 114,981 healthy controls [8]. The genetic risk score was estimated by inclusion of all available SNPs (*n* = 120,991) based on a meta-analysed by Morris et al. [17]. The meta-analytic effect size of each of the SNPs was transformed into a Z-score and multiplied by the number of copies (0/1/2) of the effect allele carried by the individual. These individual risks across all SNPs were summed to form participants' type 2 diabetes all-inclusive polygenic risk score. The calculations were done using PLINK software [37]. A more detailed description of the scoring procedure is provided elsewhere [38].

### Covariates

#### Age and sex

Sex was coded as 0 for females and 1 for males. Age was treated as a continuous variable.

#### Cognitive ability

Cognitive ability at age 70 was assessed using the Moray House Test no. 12. The test consisted of a variety of items designed to assess reasoning ability, e.g., word classification, analogies, reasoning, and spatial items [30]. Cognitive ability was treated as a continuous variable.

#### SES indicators

Highest educational qualification was classified into five categories ranging from ‘no qualification’ to ‘university degree’. Occupational class was assessed on a five-point scale ranging from ‘manual labour’ to ‘professional’ [39]. Women who reported lower occupational class than their spouse were classified according to their spouses. Both the highest educational qualification and occupational class were treated as continuous variables.

### Analyses

We fit a series of linear regression models predicting HbA1c from its polygenic risk score and five personality traits. Models 1–5 tested the main effects of each of the five personality domains, and their interactions with polygenic risk. Model 6 tested the main effects of all five personality traits taken together and their interactions with polygenic risk. Finally, we ran the same set of models, but controlling for the effects of cognitive ability and socioeconomic status. All models were fitted using R 3.1.3 [40].

## Results

Ninety-one (10.9%) participants showed HbA1c levels higher than the diagnostic cut-off value (6.5 mmol/L) that would lead to a diabetes diagnosis [41]. Polygenic risk score is known to predict the HbA1c measure in the LBC36 sample [17] and did so in our study (*β* = 0.15, *p* < .001). Participants with higher levels of HbA1c had higher neuroticism, were less open to experience and had lower agreeableness than those with lover levels (Table 1). The differences between participants with normal and heightened levels of HbA1c were significant for openness (*p* = .020) and agreeableness (*p* = .004), but not for neuroticism (*p* = .21). The full list of group comparisons is presented in Table 1.

Of personality traits, openness to experience and agreeableness were negatively correlated with HbA1c (*r* = − 0.07, *p* = 0.028, and *r* = − 0.08, *p* = 0.013, respectively). The only personality trait related to T2D polygenic risk was agreeableness (*r* = − 0.08, *p* = 0.021). The matrix of correlation between all variables in the study is presented in Table S1.

First, we tested whether personality traits moderated the expression of genetic risk for type 2 diabetes. Models 1–5 (Table 2) included the main effect of one personality trait at a time, and its interaction with polygenic risk, whilst controlling for the effects of age and sex. Lower levels of openness were related to heightened HbA1c (*β* = − 0.07, *p* = .036), but the interaction between openness and polygenic risk was not significant. Furthermore, in the model including personality trait agreeableness, lower agreeableness was related to heightened levels of HbA1c (*β* = − 0.07, *p* = .033), and the interaction between agreeableness and T2D polygenic risk was also significant (*β* = − 0.07, *p* = .033) — the nature of the interaction is considered below. The final model (Table 2) included the effects of all five personality traits: neuroticism, openness, agreeableness, extraversion and conscientiousness. In this model, both the main effects of personality traits, and the interactions between personality traits and T2D polygenic risk were comparable to those in models 1–5. The main effect of openness to experience was related to HbA1c — higher openness was associated with lower HbA1c levels (*β* = − 0.07, *p* = .040), and the interaction between openness and polygenic risk was not significant. The main effect of agreeableness was no longer significant (*β* = − 0.05, *p* = .20), although similar in magnitude to the one in Model 4. However, the interaction between agreeableness and T2D polygenic risk was still significant (*β* = − 0.08, *p* = .021). No other main effects of personality traits or interaction terms with T2D polygenic risk were significant in this model (Table 2).

The next set of models were the same as Models 1–6, but adjusted for the effects of cognitive ability at age 70, highest educational attainment, and occupational class, in addition to age and sex (Table 3). In the model that included the effects of one personality trait at a time, adjusting for these covariates attenuated the effects of openness. This time, neither the main effect of openness (*β* = − 0.04, *p* = .27), nor the interaction between openness and T2D was significant. This was not specific to the inclusion of cognitive ability or socioeconomic status indicators — including just one of the confounders at a time produced the same effect. However, both the main effect of agreeableness (*β* = − 0.07, *p* = .028) and its interaction with T2D polygenic risk (*β* = − 0.05, *p* = .010) were still significant after adjusting for the effects of IQ, educational attainment and occupational class. In the model that included all five personality traits along with the covariates (Model 6a), the main effect of agreeableness was not significant (*β* = − 0.06, *p* = .091). However, the interaction between agreeableness and polygenic risk was still significant (*β* = − 0.07, *p* = .036). Finally, the interaction between conscientiousness and diabetes polygenic risk was significant in the model including all traits and all covariates (*β* = 0.09, *p* = .015).

**Fig. 1:**
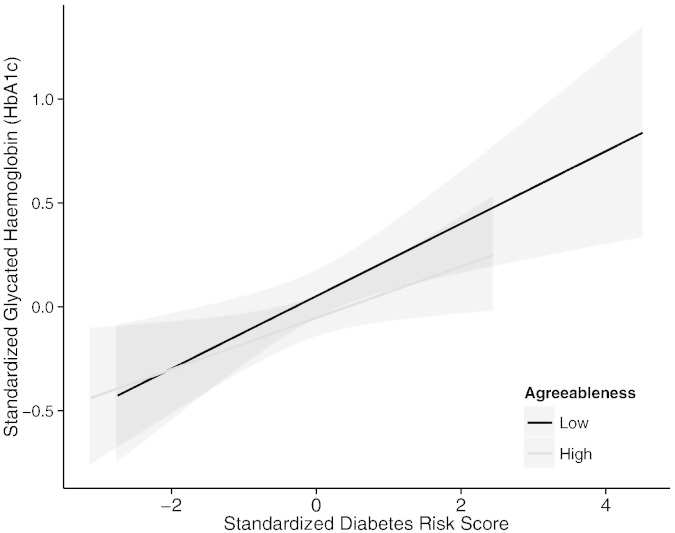
Association between HbA1c levels and genetic risk for type 2 diabetes in high and low agreeableness groups. *Note*. Shaded lines represent 95% confidence intervals. Correlations between diabetes polygenic risk and HbA1c in low and high agreeableness groups: *r*(322) = .17, *p* = .002 and *r*(331) = .13, *p* = .02, respectively.

**Fig. 2:**
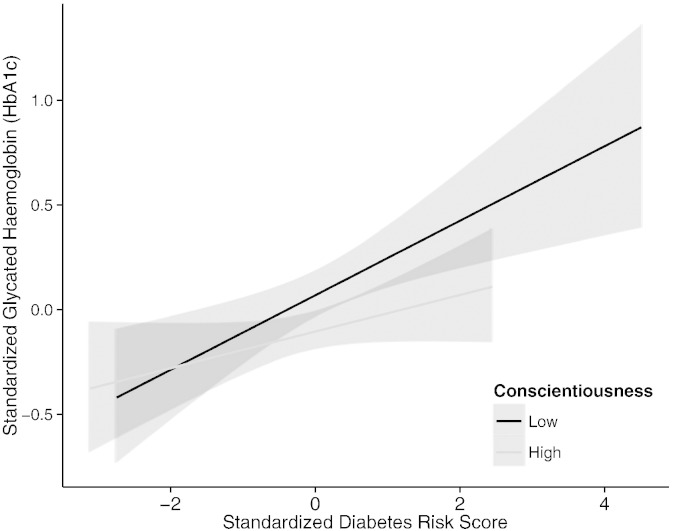
Association between HbA1c levels and genetic risk for type 2 diabetes in high and low conscientiousness groups. *Note*. Shaded lines represent 95% confidence intervals. Correlations between diabetes polygenic risk and HbA1c in low and high conscientiousness groups: *r*(382) = .17, *p* = .001 and *r*(271) = .10, *p* = .09, respectively.

To clarify the observed interactions between T2D polygenic risk and personality traits agreeableness and conscientiousness, we performed a median split of participants on the two personality traits and predicted HbA1c from T2D polygenic risk separately in the lower and higher agreeableness groups. The differential predictions are presented in Figs. 1 and 2, respectively and suggest that the polygenic risk for diabetes was more strongly associated with HbA1c levels among participants with lower levels of agreeableness, and lower levels of conscientiousness.

We ran an additional set of models to address whether the associations between personality traits and T2D polygenic risk were mediated by depression. Controlling for depression did not affect the interaction between conscientiousness and type 2 diabetes polygenic risk (*β* = 0.09, *p* = .017). However, the interaction between agreeableness and T2D polygenic risk dropped below the significance threshold (*β* = − 0.06, *p* = .051). The set of models controlling for depression is presented in full in Table S2 of Supplementary Material.

## Discussion

The current findings suggest that lower levels of openness are associated with higher levels of HbA1c. However, this association did not remain after controlling for cognitive ability, although the effect size remained similar in magnitude. Furthermore, the genetic risk for diabetes was more strongly associated with HbA1c levels among participants who were lower in agreeableness and lower in conscientiousness, though the latter was true only in models that adjusted for cognitive ability, SES indicators, and included all five personality traits.

Our finding that lower openness was related to higher levels of HbA1c is consistent with a cross-sectional study [20]. However, the link between openness and HbA1c was explained by the association between openness and cognitive ability, which was both previously reported [42] and found in the current study (*r* = .26). Cognitive ability has been associated with diabetes onset [17,43], and moderates the expression of its T2D genetic risk [17].

We also found that lower agreeableness enhanced the expression of the genetic risk for diabetes. One possible confounding mechanism is through links of personality with socioeconomic status [44]. Lower agreeableness is associated with lower levels of education, an indicator of socioeconomic status [45], which is in turn associated with poorer health in general [46], and type 2 diabetes prevalence in particular [47]. Including indicators of socioeconomic status, namely educational attainment and occupational status, attenuated the strength of the interaction between agreeableness and T2D polygenic risk, but this attenuation was very small in size (*β* = − 0.08 in the unadjusted model compared to *β* = − 0.07 in the adjusted model). On the other hand, lower agreeableness may lead to higher expression of T2D genetic risk via its associations with health harming behaviours [44] and overall unhealthy lifestyle factors [28,48]. Another mechanism may be that lower agreeableness is linked with lower trust in healthcare system and poor patient–doctor communication, which may lower the chances of diabetes symptom detection or impair their effective management [44].

The finding that the interaction between polygenic risk and conscientiousness was significant in the fully adjusted model suggests that variance unique to conscientiousness is associated with lower expression of the genetic risk for diabetes. This is in line with a previous report that found lower levels of conscientiousness in people with diabetes than in those without the disease [20]. Furthermore, conscientiousness is consistently associated with beneficial health outcomes, including longer life span [49]. This is not surprising, as conscientiousness is linked to health promoting behaviours, such as healthier diet and exercise [50] and better adherence to medical treatment [44].

The present study had some limitations. First, we were not able to distinguish between type 1 and type 2 diabetes. Given that genetic risk for type 2 diabetes is likely different from that of type 1 diabetes [51–54] and that the two forms of the disease have different mechanisms of action [55], the role of personality traits as potential moderators possibly varies between types. However, only six participants reported taking insulin. Removing these participants from the analyses did not alter present results. Second, our sample was relatively small for a genetically informative design. Thus, the results should be replicated using a larger genetically informative sample. Finally, in the present study we focused on the five personality traits as described by the FFM. It is possible that other operationalisations of the variance in human personality, like PEN [56], TCI [57], and MPQ [58] models, could yield interesting findings. However, the FFM is commonly utilised in the study of personality and health outcomes in general [44], and in the studies relating personality and diabetes mellitus in particular [20–23]. To better understand the observed relationships between personality traits and diabetes, future studies should investigate whether the associations are driven by lower level, more specific descriptors of personality, such as personality facets [59].

In conclusion, we found evidence that suggests that personality traits may moderate the effects of common genetic variants predisposing to diabetes. Potential mechanisms of these associations are lifestyle factors such as dietary habits and exercise, and general levels of health concerns.

## Conflict of interest statement

The authors declare no conflict of interest.

## Figures and Tables

**Table 1:** Descriptive statistics of the sample by HbA1c levels.

	Total	HbA1c levels		Comparisons	
Low	High	Low vs. high	
*M* (*SD*)	*M* (*SD*)	*M* (*SD*)	*t*	*p*
HbA1c	5.92 (0.74)	5.73 (0.34)	7.54 (1.04)	− 34.54	< .001
Neuroticism	17.09 (7.71)	16.97 (7.69)	18.05 (7.88)	− 2.77	.20
Extraversion	26.98 (5.90)	26.96 (5.88)	27.13 (6.16)	− 0.26	.80
Openness	26.04 (5.79)	26.20 (5.77)	24.67 (5.82)	2.39	.017
Agreeableness	33.42 (5.33)	33.60 (5.31)	31.89 (5.23)	2.90	.004
Conscientiousness	34.71 (5.99)	34.78 (5.96)	34.12 (6.23)	0.99	.32
Age	69.58 (0.85)	69.57 (0.86)	69.72 (0.79)	− 1.65	.10
Cognitive Ability	101.35 (13.76)	101.87 (13.21)	97.01 (17.17)	3.16	.002
Education	1.72 (1.29)	1.77 (1.30)	1.32 (1.14)	3.15	.002
Occupation	2.37 (0.91)	2.34 (0.90)	2.56 (0.93)	3.15	.036

		*n* (%)	*n* (%)	*X*^2^	*p*

Gender				1.30	.25
Male		358 (48.0)	50 (54.9)		
Female		388 (52.0)	41 (45.01)		

*Note*. Low HbA1c < 6.5 mmol/L, *n* = 746; High HbA1c ≥ 6.5 mmol/L, *n* = 91; Total *n* = 837. Education = highest educational attainment. Occupation = occupational class.

**Table 2:** Standardized betas (standard errors) in the models predicting HbA1c levels using type 2 diabetes (T2D) polygenic risk and personality traits.

	Model 1		Model 2		Model 3		Model 4		Model 5		Model 6	
*β* (SE)	*p*	*β* (SE)	*p*	*β* (SE)	*p*	*β* (SE)	*p*	*β* (SE)	*p*	*β* (SE)	*p*
Intercept	− 6.87 (2.65)	.009	− 6.93 (2.65)	.009	− 7.03 (2.62)	.007	− 6.64 (2.61)	.011	− 6.81 (2.67)	.011	− 6.34 (2.69)	.019
Age	0.00 (0.00)	.009	0.00 (0.00)	.010	0.00 (0.00)	.008	0.00 (0.00)	.012	0.00 (0.00)	.011	0.00 (0.00)	.020
Male vs. female	0.03 (0.07)	.69	0.04 (0.06)	.59	0.05 (0.06)	.48	0.07 (0.07)	.32	0.04 (0.06)	.50	0.07 (0.07)	.33
T2D polygenic risk	0.15 (0.03)	< .001	0.14 (0.03)	< .001	0.14 (0.03)	< .001	0.12 (0.32)	< .001	0.15 (0.03)	< .001	0.12 (0.03)	< .001
Neuroticism	0.04 (0.03)	.21									0.01 (0.04)	.89
N × T2D polygenic risk	0.05 (0.03)	.11									0.05 (0.04)	.20
Extraversion			− 0.03 (0.03)	.36							0.01 (0.04)	.76
E × T2D polygenic risk			− 0.01 (0.81)	.81							0.01 (0.04)	.80
Openness					− 0.07 (0.03)	.036					− 0.07 (0.03)	.04
O × T2D polygenic risk					0.02 (0.03)	.41					0.05 (0.03)	.12
Agreeableness							− 0.07 (0.03)	.033			− 0.05 (0.04)	.20
A × T2D polygenic risk							− 0.07 (0.03)	.033			− 0.08 (0.03)	.021
Conscientiousness									− 0.05 (0.03)	.12	− 0.04 (0.04)	.30
C × T2D polygenic risk									0.01 (0.03)	.77	0.06 (0.04)	.12

*Note*. *n* = 837; N = neuroticism, E = extraversion, O = openness, A = agreeableness, C = conscientiousness.

**Table 3:** Standardized betas (standard errors) in the models predicting HbA1c levels using type 2 diabetes (T2D) polygenic risk and personality traits controlling for cognitive ability, education and occupational class.

	Model 1		Model 2		Model 3		Model 4		Model 5		Model 6	
*β* (SE)	*p*	*β* (SE)	*p*	*β* (SE)	*p*	*β* (SE)	*p*	*β* (SE)	*p*	*β* (SE)	*p*
Intercept	− 5.96 (2.62)	.023	− 5.86 (2.62)	.025	− 6.21 (2.60)	.017	− 5.49 (2.59)	.034	− 5.64 (2.64)	.033	− 5.56 (2.65)	.036
Age	0.00 (0.00)	.019	0.00 (0.00)	.020	0.00 (0.00)	.013	0.00 (0.00)	.027	0.00 (0.00)	.026	0.00 (0.00)	.030
Male vs. female	0.02 (0.07)	.80	0.01 (0.06)	.82	0.02 (0.06)	.75	0.05 (0.07)	.48	0.02 (0.06)	.76	0.07 (0.07)	.35
T2D polygenic risk	0.14 (0.03)	< .001	0.13 (0.03)	< .001	0.13 (0.03)	< .001	0.13 (0.03)	< .001	0.014 (0.03)	< .001	0.11 (0.03)	< .001
Cognitive ability	− 0.08 (0.03)	.034	− 0.09 (0.04)	.016	− 0.07 (0.04)	.064	− 0.06 (0.04)	.11	− 0.09 (0.04)	.017	− 0.08 (0.04)	.057
Highest qualification	− 0.08 (0.04)	.010	− 0.08 (0.03)	.008	− 0.08 (0.03)	.013	− 0.09 (0.03)	.005	− 0.09 (0.03)	.007	− 0.08 (0.03)	.009
Occupational class	− 0.04 (0.04)	.37	− 0.04 (0.03)	.39	− 0.05 (0.04)	.21	− 0.04 (0.04)	.31	− 0.04 (0.03)	.35	− 0.04 (0.04)	.057
Neuroticism	0.02 (0.03)	.64									− 0.03 (0.04)	.43
N × T2D polygenic risk	0.03 (0.03)	.36									0.04 (0.04)	.31
Extraversion			− 0.04 (0.03)	.19							− 0.03 (0.04)	.50
E × T2D polygenic risk			0.01 (0.03)	.79							0.01 (0.03)	.71
Openness					− 0.04 (0.03)	.27					− 0.03 (0.04)	.35
O × T2D polygenic risk					.03 (0.03)	.36					0.05 (0.03)	.10
Agreeableness							− 0.07 (0.03)	.028			− 0.06 (0.04)	.091
A × T2D polygenic risk							− 0.05 (0.03)	.010			− 0.07 (0.03)	.036
Conscientiousness									− 0.05 (0.03)	.15	− 0.03 (0.04)	.34
C × T2D polygenic risk									0.04 (0.03)	.16	0.09 (0.04)	.015

*Note*. *n* = 812; T2D = type 2 diabetes, N = neuroticism, E = extraversion, O = openness, A = agreeableness, C = conscientiousness.
