# Supplementary material for: Do personality traits moderate the manifestation of type 2 diabetes genetic risk?
Source: J Psychosom Res. 2015 Oct;79(4):303–8. doi: 10.1016/j.jpsychores.2015.07.003 (PMC4579920; doi:10.1016/j.jpsychores.2015.07.003)
Supplement: Supplementary file 1 — Supplementary material. [file mmc1.docx]

Table S1

*Matrix of correlations between all variables in the study*

|  | HbA1c | T2D  Polygenic Risk | Cognitive  Ability | Education | Occupational Class | Depression | N | E | O | A |
| --- | --- | --- | --- | --- | --- | --- | --- | --- | --- | --- |
| T2D Polygenic Risk | 0.16^***^ | 1 |  |  |  |  |  |  |  |  |
| Cognitive Ability | -0.10^*^ | 0.01 | 1 |  |  |  |  |  |  |  |
| Education | -0.14^***^ | -0.03 | 0.46^***^ | 1 |  |  |  |  |  |  |
| Occupational Class | 0.06 | -0.02 | -0.36^***^ | -0.54^***^ | 1 |  |  |  |  |  |
| Depression | 0.09^**^ | 0.03 | -0.14^***^ | -0.10^*^ | 0.09^*^ | 1 |  |  |  |  |
| N | 0.03 | -0.02 | -0.19^***^ | -0.13^***^ | 0.13^***^ | 0.39^***^ | 1 |  |  |  |
| E | -0.03 | 0.01 | 0.00 | -0.06 | -0.01 | -0.40^***^ | -0.39^***^ | 1 |  |  |
| O | -0.07^*^ | -0.02 | 0.26^***^ | 0.33^***^ | -0.28^***^ | -0.14^***^ | -0.05 | 0.20^***^ | 1 |  |
| A | -0.08^*^ | -0.08^*^ | 0.11^***^ | 0.04 | -0.09^*^ | -0.21^***^ | -0.20^***^ | 0.20^***^ | 0.14^***^ | 1 |
| C | -0.05 | 0 | -0.02 | -0.02 | 0.00 | -0.25^***^ | -0.38^***^ | 0.33^***^ | 0.00 | 0.24^***^ |

*Note.* *** p < .001; ** p < .01, * p < .05; N = neuroticism, E = extraversion, O = openness, A = agreeableness, C = conscientiousness.

*Depression.* Depression was assessed using the Hospital Anxiety and Depression SCALE (HADS) depression score (Zigmnot & Snaith, 1983). The scale consists of seven items addressing mood states, rated on a 4-point scale ranging from “never” to “often”. The maximum score is 21, and a score of 11 and above indicates probable depression (Zigmnot & Snaith, 1983). The scale is a reliable and well-validated measure of depression (Bjelland, Dahl, Haug & Neckelmann, 2002). Only two participants in the present study had a score higher than the diagnostic cut-off of 11.

Table S2

*Standardized Betas (Standard Errors) in the models* *predicting HbA1c levels using type 2 diabetes (T2D) polygenic risk and personality traits controlling for cognitive ability, education, occupational class and depression.*

|  | Model 1 | | Model 2 | | Model 3 | | Model 4 | | Model 5 | | Model 6 | |
| --- | --- | --- | --- | --- | --- | --- | --- | --- | --- | --- | --- | --- |
|  | Β (SE) | P | B (SE) | P | B (SE) | P | B (SE) | P | B (SE) | P | B (SE) | P |
| Int Intercept | -6.02 (2.61) | .022 | -5.75 (2.61) | .028 | -6.13 (2.59) | .018 | -5.53 (2.58) | .033 | -5.77 (2.63) | .029 | -5.64 (2.65) | .033 |
| Age | 0.00 (0.00) | .018 | 0.00 (0.00) | .023 | 0.00 (0.00) | .014 | 0.00 (0.00) | .026 | 0.00 (0.00) | .023 | 0.00 (0.00) | .028 |
| Male vs. Female | 0.05 (0.07) | .48 | 0.02 (0.06) | .76 | 0.03 (0.06) | .66 | 0.04 (0.07) | .50 | 0.03 (0.06) | .67 | 0.08 (0.07) | .28 |
| T2D Polygenic risk | 0.14 (0.03) | < .001 | 0.13 (0.03) | < .001 | 0.13 (0.03) | < .001 | 0.12 (0.03) | < .001 | 0.14 (0.03) | < .001 | 0.11 (0.03) | .11 |
| Cognitive Ability | -0.07 (0.04) | .069 | -0.08 (0.04) | .052 | -0.06 (0.04) | .15 | -0.05 (0.04) | .19 | -0.07 (0.04) | .054 | -0.06 (0.04) | .11 |
| Highest Qualification | -0.08 (0.03) | .012 | -0.08 (0.03) | .011 | -0.08 (0.03) | .013 | -0.08 (0.03) | .007 | -0.08 (0.03) | .008 | -0.08 (0.03) | .012 |
| Occupational Class | -0.04 (0.04) | .40 | -0.04 (0.04) | .39 | -0.05 (0.04) | .20 | -0.04 (0.04) | .29 | -0.04 (0.04) | .31 | -0.04 (0.04) | .40 |
| Depression | 0.12 (0.04) | < .001 | 0.10 (0.04) | .003 | 0.10 (0.03) | .002 | 0.09 (0.03) | .005 | 0.11 (0.03) | .58 | 0.11 (0.04) | .005 |
| Neuroticism | -0.03 (0.04) | .35 |  |  |  |  |  |  |  |  | -0.06 (0.04) | .15 |
| N x T2D Polygenic Risk | 0.03 (0.03) | .41 |  |  |  |  |  |  |  |  | 0.04 (0.04) | .33 |
| Extraversion |  |  | -0.01 (0.03) | .95 |  |  |  |  |  |  | 0.00 (0.04) | .99 |
| E x T2D Polygenic Risk |  |  | 0.01 (0.03) | .77 |  |  |  |  |  |  | 0.01 (0.03) | .71 |
| Openness |  |  |  |  | -0.03 (0.03) | .40 |  |  |  |  | -0.03 (0.04) | .40 |
| O x T2D Polygenic Risk |  |  |  |  | 0.03 (0.03) | .33 |  |  |  |  | 0.05 (0.03) | .33 |
| Agreeableness |  |  |  |  |  |  | -0.05 (0.03) | .12 |  |  | -0.05 (0.04) | .71 |
| A x T2D Polygenic Risk |  |  |  |  |  |  | -0.04 (0.03) | .15 |  |  | -0.06 (0.03) | .051 |
| Conscientiousness |  |  |  |  |  |  |  |  | -0.02 (0.03) | .58 | -0.03 (0.04) | .42 |
| C x T2D Polygenic Risk |  |  |  |  |  |  |  |  | 0.05 (0.03) | .14 | 0.09 (.04) | .017 |

*Note.* *n* = 810; T2D = type 2 diabetes, N = neuroticism, E = extraversion, O = openness, A = agreeableness, C = conscientiousness.

**References**

Bjelland, I., Dahl, A. A., Haug, T. T., & Neckelmann, D. (2002). The validity of the Hospital Anxiety and Depression Scale: an updated literature review. *Journal of psychosomatic research*, *52*(2), 69-77.

Zigmond, A. S., & Snaith, A. P. (1983). The Hospital Anxiety and Depression Scale. *Acta Psychiatrica*, 67, 361–370.
